# Perspectives on Promoting Physical Activity Using eHealth in Primary Care by Health Care Professionals and Individuals With Prediabetes and Type 2 Diabetes: Qualitative Study

**DOI:** 10.2196/39474

**Published:** 2023-01-20

**Authors:** Yohannes Woldamanuel, Jenny Rossen, Susanne Andermo, Patrik Bergman, Linda Åberg, Maria Hagströmer, Unn-Britt Johansson

**Affiliations:** 1 Department of Health Promoting Science Sophiahemmet University Stockholm Sweden; 2 Department of Neurobiology, Care Sciences and Society Karolinska Institutet Stockholm Sweden; 3 Department of Medicine and Optometry eHealth Institute Linnaeus University Kalmar Sweden; 4 Smedby Primary Care Center Kalmar Stockholm Sweden; 5 Academic Primary Health Care Center Region Stockholm Stockholm Sweden; 6 Department of Clinical Science and Education, Södersjukhuset Karolinska Institutet Stockholm Sweden

**Keywords:** eHealth, focus groups, health care professionals, physical activity, prediabetes, primary care, qualitative research, self-management, type 2 diabetes

## Abstract

**Background:**

The trend of an exponential increase in prediabetes and type 2 diabetes (T2D) is projected to continue rising worldwide. Physical activity could help prevent T2D and the progression and complications of the disease. Therefore, we need to create opportunities for individuals to acquire the necessary knowledge and skills to self-manage their chronic condition through physical activity. eHealth is a potential resource that could facilitate self-management and thus improve population health. However, there is limited research on users’ perception of eHealth in promoting physical activity in primary care settings.

**Objective:**

This study aims to explore the perspectives of health care professionals and individuals with prediabetes and T2D on eHealth to promote physical activity in primary care.

**Methods:**

A qualitative approach was applied using focus group discussions among individuals with prediabetes or T2D (14 participants in four groups) and health care professionals (10 participants in two groups). The discussions were audio-recorded and transcribed verbatim. Qualitative content analysis was used inductively to code the data.

**Results:**

Three main categories emerged: utility, adoption process, and accountability. The utility of eHealth was described as a motivational, entertaining, and stimulating tool. Registration of daily medical measurements and lifestyle parameters in a cohesive digital platform was recognized as a potential resource for strengthening self-management skills. The adoption process includes eHealth to increase the accessibility of care and personalize the support of physical activity. However, participants stated that digital technology might only suit some and could increase health care providers’ administrative burden. Accountability refers to the knowledge and skills to optimize eHealth and ensure data integrity and security.

**Conclusions:**

People with prediabetes and T2D and health care professionals positively viewed an integration of eHealth technology in primary care to promote physical activity. A cohesive platform using personal metrics, goal-setting, and social support to promote physical activity was suggested. This study identified eHealth illiteracy, inequality, privacy, confidentiality, and an increased workload on health care professionals as factors of concern when integrating eHealth into primary care. Continuous development of eHealth competence was reported as necessary to optimize the implementation of eHealth technology in primary care.

## Introduction

The prevalence of prediabetes and type 2 diabetes (T2D) is steadily increasing worldwide [1]. Prediabetes is an intermediate stage between normal glycemia and diabetes [2] and a risk factor for progression to T2D and cardiovascular diseases [3]. Previous randomized controlled studies, including diabetes prevention programs, have shown that lifestyle therapy prevents or delays T2D and improves cardiometabolic markers [4]. T2D is a highly heterogeneous disease and includes people with different clinical characteristics, disease progression, drug response, and risk of long-term complications [5] (ie, macrovascular and microvascular complications [6]). These long-term complications imply that persons with T2D must adhere to a lifelong healthy lifestyle and access to medical care management [7]. An evidence-based sustainable effort is crucial at the individual and health care system levels to prevent or delay these complications [8].

Emphasis should be given to primary care as it is considered an ideal setting for supporting lifestyle changes [9]. A well-designed self-management support system is needed to enable patients to deal with this lifelong challenging disease [10]. Developing person-centered diabetes self-management skills and obtaining the support needed to facilitate knowledge and decision-making skills are necessary for diabetes management [11]. However, the current health care infrastructure faces challenges due to the sustained shortage of health care professionals (HCPs) and a significant increase in the prevalence of T2D [12].

Physical activity is recommended as a critical self-management activity in individuals with T2D [13]. The positive impact of physical activity on glycemic control, insulin sensitivity, and other diabetes-related health complications is evident [14]. Yet, providing support for physical activity that matches the current health status and physical capabilities of people with T2D is challenging [15]. Studies have shown that physical activity among adults with T2D is generally low [16-18]. Thus, promoting physical activity in persons with T2D is necessary to improve their quality of life [19].

eHealth is a potential resource in the health care system for facilitating self-management support, such as continuously recording the health status with countermeasure responses, allowing self-monitoring options, and providing information that helps patients to make informed decisions related to their chronic condition [20,21]. According to the World Health Organization, eHealth is about using digital tools and sharing information digitally to achieve and maintain a high level of health [22]. Technology (ie, hardware, devices, and software) should be customized based on the patient’s needs, desires, skill level, and availability of devices [23]. The use of eHealth and mobile health interventions is a cost-effective approach that reaches many individuals with a high level of engagement in the short-term [24,25]. eHealth interventions also have had a considerable impact on physical activity and healthy eating. However, eHealth interventions have not shown a long-term effect and have not been applied to large-scale implementations [26]. The implementation of eHealth might depend on consumers’ satisfaction with using communication platforms efficiently and sustainably with their health care providers [27].

eHealth has been applied as a self-management tool option in current health care practice. It is also used to create an effective communication channel between patients and HCPs in diabetes disease control [28,29]. There is an inefficiency in promoting physical activity by the HCPs during their encounters with their patients in primary care [30,31]. Therefore, there is a clear need to identify strategies to integrate eHealth tools to promote physical activity in primary care settings. A review focusing on physical activity counseling to patients in primary health care has shown the potential of using eHealth tools and highlights the importance of identifying facilitators of and barriers to the usability of eHealth tools in the setting [32]. Previous researchers have noted a high rate of attrition in eHealth interventions. Explanations to the problem of the high dropout rate are required to find solutions [33,34]. Few studies have investigated the perspective of individuals living with chronic diseases on the use of eHealth and its integration with person-centered care [35-37].

Therefore, this study aimed to explore the perspectives of HCPs and individuals with prediabetes and T2D on eHealth to promote physical activity in primary care, which may provide insight into how eHealth can be optimized to promote physical activity.

## Methods

### Research Design

An exploratory descriptive qualitative study design was applied. The data were collected using semistructured focus group discussions to explore the participants’ experiences and perceptions [38]. COREQ (Consolidated Criteria for Reporting Qualitative Research) was used to notify the critical aspects of the research method (Multimedia Appendix 1).

### Recruitment and Participants

Two groups of participants with different positions regarding their contact with the primary care center, care providers (HCPs), and patients were eligible. A total of 53 individuals were asked to participate in the study.

HCPs were eligible if they were working in primary care. The recruitment locations were six primary care centers in Stockholm (an urban area) and two in villages in the south of Sweden (a rural area). Convenience sampling was used to recruit the participants who were willingly available. A total of 18 HCPs were approached by author JR face to face or by telephone in January 2019. They were all willing to participate in the study, but 8 participants canceled due to a lack of time and scheduling conflicts. A total of 10 female HCPs took part in the focus group discussion; 2 of them were physicians, and 8 were diabetes specialist nurses.

The second group consisted of patients with prediabetes and T2D. This group was eligible if registered as patients in primary care centers and diagnosed with prediabetes or T2D. A total of 34 individuals were approached face to face by authors JR and LÅ, and were willing to participate in the study. This group was divided further into the Sophia Step Study (SSS) group and the non-SSS group. Participants from the SSS were recruited using a purposeful sampling method to identify individuals with specific eHealth experiences. They had previously participated in a 2-year intervention program using pedometers and a website to register their daily steps [39]. A total of 14 patients were included in the study, 2 of whom were diagnosed with prediabetes, and 9 patients participated in the SSS intervention. Most of the participants’ cancelations were due to a lack of time and personal issues.

### Data Collection

The data were collected in February-April 2019. JR and LÅ conducted a total of six semistructured focus group discussions. Two focus group discussions with the HCPs, with 5 participants in each group, and four group discussions with SSS and non-SSS participants. The focus group discussions with patients had 2 to 6 participants per session. The focus group discussions were held in Swedish and lasted from 55 to 82 minutes. All the focus group discussions were conducted in rooms with a group discussion–friendly environment at the primary care centers. Interviews were audio-recorded, and field notes were made during the discussions.

Participants filled out a brief questionnaire asking for demographics and experiences on the use of eHealth at the beginning of each group session. At the start of the group discussion, JR introduced the purpose of the study and the procedural activities during the session. Information was also given about recording focus group discussions using a digital recorder and that observational notes would be taken to capture the context of the discussions by LÅ. The focus group discussions were based on semistructured interview guides (Multimedia Appendix 2). Open-ended questions were used to explore patients’ and HCPs’ perspectives regarding eHealth technology to promote the physical activity of patients with diabetes in a primary care context.

### Data Analysis

Audio recordings were transcribed verbatim by a professional transcriber. The transcripts were analyzed using inductive content analysis [40]. A procedure consisting of five phases was developed before the study began to enhance the trustworthiness and credibility of the data analysis. In the first phase, authors U-BJ and YW independently checked for the accuracy of the transcribed text against the audio-recorded files. In the second phase, U-BJ, JR, and YW read and repeatedly listened to the transcribed material to better understand the content and identify meaning units. The meaning units were condensed in the third phase without losing the original meaning. After that, the research group (JR, U-BJ, and YW) discussed the differences in the selected meaning units and reached a consensus. In the fourth phase, the research group, including author SA, started extracting the meaning units and assigning codes. The researchers thoroughly discussed the meaning units to identify differences and similarities of the codes. Lastly, the codes were examined for relations, sub-merged from meaning units, and grouped under potential subcategories. These subcategories were grouped into categories and appropriately named after reaching a consensus. Quotes were selected to represent the variations of the participant groups. Finally, a professional translator translated the results and quotations from Swedish to English.

Descriptive statistics were calculated and summarized for demographic characteristics using SPSS version 27.0 for Windows (IBM Corp). Data are presented as mean (SD) or number (percentage) as appropriate. The qualitative data were organized and analyzed manually; no software application was used.

### Ethics Approval

The study was approved by the Regional Ethical Review Authority in Stockholm (2018/28-31/2). All invited participants gave informed written consent to participate in the study, and it was performed according to the Helsinki Declaration [41].

## Results

### Participants

The mean age of the 14 patients was 69 (SD 9.5) years, 71% (n=10) were males, and 86% (n=12) had T2D. More than half of the patients used different eHealth technologies privately, such as smartphones, computers, blood glucose meters, and activity bracelets. In connection with the health care system, patients used smartphones and blood glucose meters at higher percentages than other tools (Tables 1 and 2). The HCPs were all female and had a mean age of 49 (SD 12.3) years. All HCPs had experience with eHealth technologies (computers, blood glucose meters, pedometers, web-based guides) in the workplace in connection with patients at primary care centers (Table 3).

The analysis from the focus group discussions revealed three main categories and nine subcategories representing the different perspectives on eHealth to promote physical activity (Textbox 1).

**Table 1:** Characteristics of individuals with prediabetes or type 2 diabetes (n=14).

	Patients
Age (years), mean (SD)	69 (9.5)
Men, n (%)	10 (71)
Type 2 diabetes, n (%)	12 (86)
University education, n (%)	7 (50)
Retired, n (%)	11 (79)
Participants of Sophia Step Study, n (%)	9 (64)
Participants from Stockholm, n (%)	9 (64)

**Table 2:** Use of eHealth of individuals with prediabetes or type 2 diabetes (n=14).

Use of eHealth (yes)	Privately, n (%)	In contact with health care, n (%)
Smartphone	12 (86)	12 (86)
Computer	9 (64)	6 (43)
Tablet	5 (36)	2 (14)
Blood glucose meter	10 (71)	8 (57)
Pedometers	8 (57)	2 (14)
Exercise app	4 (29)	3 (21)
Activity bracelet	13 (93)	0 (0)

**Table 3:** Characteristics of health care professionals (n=10).

		Health care professional participants
Age (years), mean (SD)		49 (12.3)
Women, n (%)		10 (100)
Participants from Stockholm		6 (60)
**Profession, n (%)**		
	Nurse	8 (80)
	Physician	2 (20)
**Specialization, n (%)**		
	Diabetes nurse	4 (40)
	District nurse	4 (40)
	Specialized physician	2 (20)
**Use of eHealth in the workplace (yes), n (%)**		
	Smartphone	4 (40)
	Computer	8 (80)
	Tablet	0 (0)
	Blood glucose meter	9 (90)
	Pedometer	5 (50)
	Exercise app	3 (30)
	Activity bracelet	1 (10)

**Categories and subcategories on the promotion of physical activity.**

**Utility**

- Motivating means
- Cohesive platform
- Social support

**Adoption process**

- Transition to personalization
- Not suitable for everyone
- Adaptation

**Accountability**

- Digital skills support
- Confidentiality
- Liability

### Utility

Utility refers to the usefulness of eHealth as a motivational and multifunctional tool, and as a facilitator of social interactions.

#### Motivating Means

eHealth products and tools were described as motivational, entertaining, and stimulating as a support for physical activity. Participants used eHealth technologies through websites as information sources offered by health care authorities (eg, to acquire information about their illness and tips and advice on lifestyle modification). They also mentioned eHealth technologies that assess personal metrics including daily physical activity and blood sugar levels as well as by adding features on goal setting. The ability to compare results with yourself and against others was considered fun and uplifting.

But sometimes you go down and sometimes up [registration of steps on a website] and there was such an intoxication, a real joy. This week I’m the best. Next week may not be as good.

SSS patient, urban area

Here, if you set reasonable goals, they can achieve and come up with something positive instead of working randomly.

HCP, urban area

#### Cohesive Platform

Participants described the possibilities of improving the self-management of physical activity from primary care with the help of digital technology. Combining services (eg, daily clinical measurements and lifestyle parameters in a cohesive platform) was seen as an opportunity by patients and HCPs. According to the participants, this would simplify reviewing and examining the characteristics of different metrics. Such a multifunctional platform was suggested as a guide to determine how behavior and health outcomes are interrelated. They also considered it a functional tool in supporting patients to be physically active if, for example, educational games, rewards, and reminders could be built into the platform.

I would like to see a common portal or app in which pedometers are located, where you combine a pedometer with blood sugar measurements during the day.

non-SSS patient, urban area

HCPs also reported that technology could be used as an educational tool to improve their practice in supporting the self-management of physical activity. Tracking patients through a cohesive platform could help evaluate patients’ self-management and provide rewards and encouragement. In addition, HCPs believed that one could better prepare for visits if the patient’s metrics were made available to the HCP in advance or if the health care system could use a web-based form linked to the medical record. The patients could also see an opportunity to participate in the care process by evaluating their activity level and development with HCPs to receive feedback by sharing their data in advance. The patients stated that this might increase the participation of patients in disease management.

That during the meeting you do an evaluation. I think that health care providers find out how far you have walked and how much you usually walk. It is important that the health care providers evaluate how the patient uses these aids (e-health tools) and if so, what do they show.

SSS patient, rural area

#### Social Support

The participants were optimistic about using a digital communication platform for groups living with T2D. Patients stated that sharing experiences among individuals with the same disease could create mutual encouragement and support. It was also suggested that meeting new people with a common interest could add value through group activities (eg, real-time chat and competition-integrated features).

It's always fun to meet new people with novel ways of thinking.…it's more about common interests. It's not about the physical part; rather, it's about having a common interest.

SSS patient, urban area

Patients suggested that creating a functional digital group might be challenging for security reasons, especially if HCPs could not have accountability in facilitating the platform to avoid potential harassment and keep track of what is said. The HCPs doubted that they would have time to take the role of a moderator who facilitated and guided the groups. However, they indicated that patients should conduct and moderate the group discussions.

There are patient associations where we are not involved or do not have any responsibility, so that it could be an online patient group. But then they have to appoint someone in charge of what is being said.

HCP, urban area

I think you should perhaps get user details and passwords via your diabetes nurse so you can log in there.

SSS patient, urban area

### Adoption Process

The adoption process refers to the challenges of ensuring individualization, creating equal opportunity, and finding the right balance in the use of eHealth.

#### Transition to Personalization

The participants reflected on the idea that implementing new eHealth tools in primary care may facilitate increasing the accessibility and personalizing the support of the physical activity. Whereas the HCPs mentioned that care might be flexible and easily accessible using digital tools and services, eHealth designs should be tailored to individual needs and preferences.

I think that in the future, the focus will be on how to develop the care provision, and you [as a clinic] put in what you want so that it can be personalized to the person at hand.

HCP, urban area

HCPs also mentioned that work routines with personalized approaches using eHealth tools can be challenging if more technical skills are also necessary. It can also lead to many administrative and time-consuming tasks overseeing vast amounts of data and increasing patient contact.

But, of course, it will hopefully give an objective image of how the patient moves. However, there can be a lot more contacts, and more administrative work if you get data that come in that you don't want to handle...

HCP, rural area

#### Not Suitable for Everyone

HCPs and patients emphasized that technology and eHealth are not suitable for everyone because of language difficulties, costs, and different technological habits between age groups. Consequently, the participants expressed concern about increasing the risk of inadvertently creating unequal care in the community.

...equal care for everyone, but it will not be so when you use apps and mobile phones. Some groups will disappear, partly because of language challenges and partly because of age , or because they don't own a mobile.

HCP, urban area

But then it's a little different how people get used to this. Older people, such as you and me, can have difficulties when it comes to technology

non-SSS patient, rural area

Participants expressed that it can be challenging for some to use eHealth technology if they have not used it earlier and must rely on the help of others. Some patients preferred using traditional paper forms or a diary for their metrics.

I completely agree that it must not replace the physical meetings, but it can be an accompaniment, and then it can be an advantage.

HCP, urban area

#### Adaptation

Personal motivation was considered a crucial factor in using eHealth, and it was vital to find the right balance in its use. The HCPs highlighted that there could be an inconvenient situation if some patients do not want or are not motivated to use digital tools to increase physical activity. It could be challenging to manage if the individual becomes stressed either by excessive interactions or an unexpected malfunctioning of the technology. One drawback of introducing eHealth could be that some patients may agree to use digital tools only to please the HCPs.

I think, above all, the technical aids are very good to have, but if you don't have the right attitude to take care of yourself, it doesn't matter how many technical aids you have.

non-SSS patient, urban area

So, it can only be a stressor...Then maybe some fill in [register the daily steps] just to make us happy and satisfied.

HCP, urban area

HCPs were concerned that using eHealth technology could increase screen time, adversely affecting the daily level of physical activity. It could also increase patients’ dependency on HCPs rather than enhanced self-management behavior.

...you can constantly measure and send messages to your doctor or diabetes nurse, or some people could become more dependent on advice and support in the app and thus end up taking less responsibility for their care...

HCP, rural area

### Accountability

Accountability refers to authority agencies being responsible for digital skills development and integrity and security concerns.

#### Digital Skills Support

Participants stressed that the rate of technological development is high, which could require continuous technical skills development for participants. New eHealth services or digital tools might be particularly challenging for older adults unless the designs and features are adapted to this age group. Accordingly, the participants stated that eHealth products and services should be clear and straightforward. The participants pointed out the need for knowledge and skills to optimize eHealth. In addition, they noted the need for informative and well-designed instructions for digital tools (tutorials).

...If it concerns e-health, it should be easily accessible and that you get knowledge about how it works, that is, education.

non-SSS patient, rural area

#### Confidentiality

The participants specifically highlighted that the integrity and security of physical activity and health data must be ensured while using eHealth services and digital tools. The patients expressed the importance of data transfer and exchange among HCPs. However, they underscored the need for a secure and safe platform for accessing personal data among HCPs and other providers.

...then there are privacy rules and things like that as well, but maybe it [personal data] should be available to only doctors and a few others.

SSS-patient, urban area

#### Liability

Participants agreed on the importance of a credible source of information about physical activity. They stated that the sources need to be credible and scientific and provide adequate knowledge. They felt that the companies in charge of developing eHealth technology should be accountable for building trust and harmony. They also addressed the importance of critically analyzing new workflow procedures and conducting assessments to use health care resources effectively. It was proposed that eHealth and health care authorities take the overall responsibility for new eHealth services and digital tools.

Yes, but it generally feels like the entrepreneurs are responsible for the development...the e-health authorities would have been fantastic, if it is they who pick up...and follow the development.

HCPs, urban area

## Discussion

### Principal Findings

This study focuses on exploring the perspectives of HCPs and patients on promoting physical activity using eHealth technology in primary care. The findings of the focus group discussions revealed three main categories: utility, adoption process, and accountability.

The category utility was built on the subcategories motivating means, cohesive platform, and social support. The participants described how eHealth technologies with a cohesive platform design could be a source of motivation and social networking for patients. In general, participants showed positive perspectives on the opportunities and usefulness of eHealth technologies to promote physical activity in primary care. Similarly, a recent review found that consumers see opportunities to use eHealth to promote physical activity and healthy dietary behaviors. However, the study elicited that several points need to be considered to optimize eHealth tools [42], which includes considering a logical and practical approach rather than pure theoretical principles [32].

The shared perspectives of participants in this study were that eHealth technologies have the potential to support assessing personal metrics and stimulating users to reflect upon them. These strategies may boost people’s motivation to change their level of physical activity and maintain it for the long-term. Similarly, a study exploring patients’ perspectives on a digital lifestyle intervention showed the importance of working on the possibility of tracking the changes, setting goals, and having tailored information to enhance the motivation and acceptability of digital health intervention support [43]. Thus, eHealth technology was seen as a motivational means of developing a personal action plan and assessing the level of achievement toward one’s goal.

Moreover, participants felt that eHealth technologies could facilitate the opportunities to have a cohesive platform for combined services to understand the relationship between behavioral changes and the body’s physiological responses. Whelan et al [44] explored the level of engagement in individuals with prediabetes using real-time feedback on their physical activity and glucose level. The authors showed that the participant’s level of engagement increased and changed their physical activity level due to real-time feedback and recognizing the link between behavior and the act on the body. As demonstrated in this study, identifying the physiological responses of being physically active might help in understanding how the body functions and stimulate the patients’ level of engagement.

In this study, having a digital communication platform for the patients to interact with their peer group was seen positively. The participants stated that the platform could create an opportunity to share experiences and deal with psychosocial problems. A cross-sectional study showed that the diabetes online community benefited from peer health experiences as a complementary resource for diabetes self-management information to enhance health literacy [45]. However, participants in our study were concerned about the potential hazards (eg, misinformation) and risk of intimidation in eHealth communication platforms (social media, blogs, discussion boards, etc).

The HCPs and patients expressed conflicting views as to who should host these social platforms. The patients believed that, to ensure efficient use, HCPs should serve as the moderator. In contrast, HCPs felt patients should take on the role of the moderator. A previous study noted that the type of social support might influence the level of engagement in eHealth in persons with diabetes [46]. The study showed that both professional and nonprofessional (friends, peers, families) social support positively impacted a person’s use of eHealth technology. However, the study also showed that patients’ private networks either facilitated or hindered the use of mobile technology for self-management [46]. The explanation might be that the level of engagement may depend on the level of supportive or unsupportive behavior among nonprofessionals. Therefore, Petroviki and Zivkovic [47] emphasized the importance of evaluating patients’ readiness and capability to handle information on social media by the HCPs to minimize the risk of misinformation and confidentiality and privacy concerns.

The second category, adoption process, was divided into three subcategories (transition to personalization, not suitable for everyone, and adaptation). eHealth technology was believed to promote person-centered care by facilitating the partnership between the patient and HCPs. A qualitative study exploring the views of different stakeholders found that the integration of person-centered care with eHealth services in primary care settings strengthens the partnership between caregivers and patients [48]. However, the HCPs in our study were concerned about the imbalance between the increased accessibility and a personalized approach and managing managerial and time-consuming tasks. A similar concern was discussed in a study about the significance of examining nurses’ workload in the integration process of eHealth services in primary health care [49].

In this study, participants raised some issues concerning the suitability of eHealth for everyone. HCPs were also concerned that patients might increase their dependency on HCPs and might not always be motivated to use eHealth technologies for disease management. Conversely, they felt that some patients might overly engage in eHealth technology and raised the possibility that the eHealth implementation process might require modification regarding health care workflow. Samarasinghe and Miras [50] considered the versatility and popularity of digital platforms in diabetes prevention interventions; however, emphasis should be given to widening opportunities at the population level with good quality and at low cost without ignoring face-to-face interaction. Moreover, a study assessing diabetes management using remote monitoring technology stressed the importance of identifying determinants that activate and engage patients in their care [51].

Our findings showed that adapting eHealth technology could increase the risk of disregarding certain groups of people (eg, those who do not use eHealth technology because of language difficulties or cost, or who have a low level of technological skills). In addition, our participants suggested that if digital inequalities could not be resolved, traditional care in combination with eHealth services should be anticipated. A review confirmed our finding that digital inequalities might occur among specific patient groups. However, the demand for an improved and advanced application to improve digital equality in eHealth services might be compelling [52]. Determinants of telemedicine use among different subgroups were, for example, being young, having a high educational level, having a higher income, and being born in Sweden. Therefore, particular consideration for people with low use of eHealth should be a priority in policy-making [53].

The accountability category included three subcategories: digital skills support, confidentiality, and liability. This category highlights the need for continuous development in the use of eHealth technology in terms of digital skills support and enhanced confidentiality. Participants were concerned about their ability to optimize technical skills on eHealth technologies for promoting physical activity. They expressed a need for clear and straightforward eHealth products and services customized for diverse groups. It was noted that the ever-changing technological progress might need a rigorous technical design and require an introduction and educational program for users. Several studies have confirmed that continuous training and proper support of eHealth services in primary care create a simplified workflow and optimal interaction between caregivers and patients [48,49,54]. In addition, eHealth services might be attainable if the integration process can accommodate staff, patient flow, and the health care data system [55].

The participants mentioned uneasiness about the integrity and confidentiality of the storage and exchange of personal data on physical activity and health. They stated that the responsibility of securing the integrity and confidentiality of the collected data should be given to eHealth and health care governmental authorities. A recent review addressed eHealth system security and privacy concerns. The review showed that the current solutions have been promising but are still inadequate because of the complexity of health systems in advanced health care services [56]. Therefore, HCPs and patients need to possess knowledge and skills to safely exchange data and secure the integration with other eHealth systems.

The focus group interviews of this study were done before the outbreak of the COVID-19 pandemic. This period might have impacted the participants’ perspectives as the consumption of digital health in primary care settings likely increased during and after the pandemic. On the contrary, a review summarizing the role of eHealth, telemedicine, or telehealth in delivering health care services during the COVID-19 pandemic showed that there was still inconsistency in the evidence on the provision of eHealth services to patients with chronic conditions [57].

### Methodological Discussion

One strength of our study was the heterogeneity of the participants. The sample includes both genders, people with prediabetes or T2D, varied experiences of using activity trackers, people from different geographical locations (urban and rural), and HCPs from several primary care contexts. Such diversity ensures a broader perspective when exploring the needs and preferences of eHealth to promote physical activity. A researcher (JR) led each focus group and engaged in the data-collecting process to maintain a higher level of consistency and avoid discrepancies. The focus group discussions of HCPs and patients were done independently. This approach created an environment for both groups to express their views freely and without reservation since the notion of power imbalance was minimized.

The trustworthiness of this study was enhanced throughout the analysis process according to Graneheim and Lundman [58]. Two researchers (U-BJ, YW) checked and rechecked the data before and during the analysis stage to confirm the data analysis outcome. It is also a strength that the two researchers were not involved in the facilitating role of the focus group, thus avoiding bias concerning the interpretation of emerging data. The process of condensation, coding, and agreeing on the categorization was made in close collaboration within the research group, which assured the credibility of the findings [58]. For instance, the researchers had different preunderstandings due to different professional backgrounds and research experiences that helped avoid unconsciously creating biases. Four of the authors (U-BJ, JR, SA, and YW) read the complete transcribed material, enabling a full picture of the content.

A limitation of the study was the use of a convenient sampling method to recruit HCP participants. Therefore, this study did not include the broader target group’s perspective and experiences, and caution needs to be taken with the generalizability of the study results. Another limitation of this study is that the group discussions were done before the outbreak of the COVID-19 pandemic, and participants might have changed perspectives on the use of eHealth after the pandemic. One of the focus group sessions included only 2 participants, which could be considered a weakness. However, the session developed into an 82-minute conversation between 2 persons, adding rich data. These issues might affect the transferability of the findings to other diabetes populations and groups of HCPs.

### Conclusions

People with prediabetes and T2D and HCPs positively viewed an integration of eHealth technology in primary care to promote physical activity. A cohesive platform using personal metrics, goal setting, and social support to promote physical activity was suggested. This study identified eHealth illiteracy, inequality, privacy, confidentiality, and an increased workload on HCPs as factors of concern when integrating eHealth into primary care. Continuous development of eHealth competence was reported as necessary to optimize the implementation of eHealth technology in primary care.

## Appendix

Multimedia Appendix 1 COREQ (Consolidated Criteria for Reporting Qualitative Research).

Multimedia Appendix 2 Interview guide.

## Abbreviations

COREQ: Consolidated Criteria for Reporting Qualitative Research
HCP: health care professional
SSS: Sophia Step Study
T2D: type 2 diabetes
