# Supplementary material for: Perspectives on Promoting Physical Activity Using eHealth in Primary Care by Health Care Professionals and Individuals With Prediabetes and Type 2 Diabetes: Qualitative Study
Source: JMIR Diabetes. 2023 Jan 20;8:e39474. doi: 10.2196/39474 (PMC9947818; doi:10.2196/39474)
Supplement: Multimedia Appendix 2 [file diabetes_v8i1e39474_app2.docx]

**Appendix 1.**

**Interview guides for the focus group discussions with individuals with pre- or type 2 diabetes and health care professionals**

1. **Focus group interview guide for people with pre- or type 2 diabetes (participants from the Sophia Step Study)**

**Introduction**

1. The moderator and the assistant will introduce themselves to the participants. Followed by presenting the study purpose, structure, and method.
2. The participants will have information about the principles and consent to participate in the study (i.e., information on free will participation and approval of withdraw at any time). They will be given assurance of security and the confidentiality of the data.
3. The focus of the discussion will be presented, which entails self-management of physical activity support using e-health in primary health care encounters.
4. The participants will present themselves to the group.
5. The ground rules for each focus group during the meeting will be presented to the participants. The meeting will start after the participants agree on the rules.
6. The moderator will define e-health and explain what services and products are included. Definition according to the Swedish national board of health and Welfare: “E-health is about using digital tools and sharing information digitally to achieve and maintain a good level of health.”

**Comprehensive questions about digitization in primary care**

1. What experience do you have regarding with using e-health technology during your contact with primary health care?
2. What do you generally think about how to use e-health technology in primary care?
3. What benefits do you think e-health technology could have for you as a patient in primary care?
4. What limitations do you think e-health technology could have for you as a patient in primary care?

**Specific questions for participants on the use of technology and e-health in the self-management support for physical activity (participants from the Sophia Step study)**

1. What are your experiences using pedometers and the website (steg.se) during the intervention periods in the Sophia Step Study?

How would you prefer e-health to be in terms of developing the design for the future?

1. What perceptions do you have regarding e-health and wearable technology that could be used in supporting self-management of physical activity?
2. What is your view on the design of e-health and wearable technology in supporting you with self-management of physical activity?
3. What care situations do you find suitable in e-health technology to support self managemnt of physical activity?
4. What is your perspective on how e-health technology support should be done during the health care team's follow-up for the self-management of physical activity?
5. What do you think about having digital health care contacts with health care workers: For example, video meeting through Web links to support self-management of physical activity?
6. What is your view on joining an independent contact group through social media platforms to share experiences among peers?
7. What are the advantages and disadvantages of e-health and wearable technology in supporting self-management of physical activity?
8. What are the advantages and disadvantages of using e-health and wearable technology for you as a primary health care patient?

**Closing questions**

1. Are there any further discussions that might be relevant to include?
2. What important things did you learn from the discussion today?
3. **Focus group interview guide for people with pre- or T2D (Participants not in the Sophia Step Study)**

**Introduction**

1. The moderator and the assistant will introduce themselves to the participants. Followed by presenting the study purpose, structure, and method.
2. The participants will have information about the principles and consent to participate in the study (i.e., information on free will participation and approval of withdraw at any time). They will be given assurance of security and the confidentiality of the data.
3. The focus of the discussion will be presented, which entails self-management of physical activity support using e-health in primary health care encounters.
4. The participants will present themselves to the group.
5. The ground rules for each focus group during the meeting will be presented to the participants. The meeting will start after the participants agree on the rules.
6. The moderator will define e-health and explain what services and products are included. Definition according to the Swedish national board of health and Welfare: “E-health is about using digital tools and sharing information digitally to achieve and maintain a good level of health.”

**Comprehensive questions**

1. What are your general impressions about using e-health and wearable technology in primary care?
2. What experiences do you have withe using of e-health technology during your contact with primary health care?
3. What benefits do you think e-health technology could have for you as a patient in primary care?
4. What limitations do you think e-health technology could have for you as a patient in primary care?

**Specific questions for participants on the use of technology and e-health in self-management support for physical activity (participants not in the Sophia Step study)**

1. Today e-health and wearable technology could support self-management of physical activity. How would you prefer these technologies to be in terms of developing the design for the future?
2. What perceptions do you have regarding activity measuring technology and e-health used to support your self-management of physical activity?
3. What are your views regarding the design of e-health and wearable technologies to support your self-management of physical activity?
4. What care situations do you find suitable in e-health technology to support self-management of physical activity?
5. What is your perspective on how e-health technology support would be done during the health care team's follow-up for self-management of physical activity?
6. Are there any disadvantages in using e-health and wearable technology for you as patients in primary care?
7. What perceptions do you have about peer training or group meetings to support self-management of physical activity?
8. What is your view on joining an independent contact group through social media platforms to share experiences among peers?
9. What do you think of the advantages and disadvantages of e-health and wearable technologies as a support of self-management of physical activity?
10. What are the advantages and disadvantages in the use of e-health and wearable technology for you as a primary healthcare center patient?

**Closing questions**

1. Are there any further discussions that might be relevant to include?
2. What important things did you learn from the discussion today?
3. **Focus group interview guide with diabetes specialist nurses and primary care physicians**

**Introduction**

1. The moderator and the assistant will introduce themselves to the participants. Followed by presenting the study purpose, structure, and method.
2. The participants will have information about the principles and consent to participate in the study (i.e., information on free will participation and approval of withdraw at any time). They will be given assurance of security and the confidentiality of the data.
3. The focus of the discussion will be presented, which entails self-management of physical activity support using e-health in primary health care encounters.
4. The participants will present themselves to the group.
5. The ground rules for each focus group during the meeting will be presented to the participants. The meeting will start after the participants agree on the rules.
6. The moderator will define e-health and explain what services and products are included. Definition according to the Swedish national board of health and Welfare: “E-health is about using digital tools and sharing information digitally to achieve and maintain a good level of health.”

**Comprehensive questions**

1. What are your general impressions about using e-health and wearable technology in primary care?
2. What experiences do you have with using of e-health and wearable technology while caring for patient with pre-or T2D in primary health care?
3. What benefits do you think e-health technology could have for you as a health care provider in primary care?
4. What limitations do you think e-health and wearable technology could have for you as a health care provider in primary care?

**Specific questions**

1. What is your view on how e-health and wearable technology can be used to support the self-management of physical activity in people with pre- or T2D?
2. What are your perceptions of how e-health and wearable technology can be designed to support self-management of physical activity?
3. Can there be any ethical aspects of using e-health and wearable technology in primary care?
4. What care situation do you find suitable in e-health technology to support self-management of physical activity?
5. What is your perspective on how e-health technology support would be done during the follow-up of patients for self-management of physical activity?
6. What is your perspective on how e-health technology support would be done during the follow-up of patient: For example, video meetings via web links to support physicala activity self-managements?
7. What is your view on patients being offered an independent contact group through social media platform to share experiences about self- management of physical activity?
8. What are the advantages and disadvantages of e-health and wearable technology as a support for self-management of physical activity?
9. What are the advantages and disadvantages of e-health and wearable technology for you as a health care provider in a primary health care r?

**Closing questions**

1. Are there any further discussions that might be relevant to include?
2. What important things did you learn from the discussion today?
